# Crosslinked Chitosan Binder for Sustainable Aqueous Batteries

**DOI:** 10.3390/nano12020254

**Published:** 2022-01-14

**Authors:** Luca Bargnesi, Federica Gigli, Nicolò Albanelli, Christina Toigo, Catia Arbizzani

**Affiliations:** Department of Chemistry “Giacomo Ciamician”, University of Bologna, Via F. Selmi 2, 40126 Bologna, Italy; luca.bargnesi2@unibo.it (L.B.); francesca.gigli2@studio.unibo.it (F.G.); nicolo.albanelli2@unibo.it (N.A.); christina.toigo2@unibo.it (C.T.)

**Keywords:** chitosan, water-soluble binder, aqueous Na ion

## Abstract

The increased percentage of renewable power sources involved in energy production highlights the importance of developing systems for stationary energy storage that satisfy the requirements of safety and low costs. Na ion batteries can be suitable candidates, specifically if their components are economic and safe. This study focuses on the development of aqueous processes and binders to prepare electrodes for sodium ion cells operating in aqueous solutions. We demonstrated the feasibility of a chitosan-based binder to produce freestanding electrodes for Na ion cells, without the use of organic solvents and current collectors in electrode processing. To our knowledge, it is the first time that water-processed, freestanding electrodes are used in aqueous Na ion cells, which could also be extended to other types of aqueous batteries. This is a real breakthrough in terms of sustainability, taking into account low risks for health and environment and low costs.

## 1. Introduction

The increasing penetration of renewable energy sources in the global panorama of energy production needs the support of large-scale stationary energy storage systems for improving grid quality, stability, and efficiency. The electrochemical energy storage systems have been demonstrated to be the most versatile to this purpose. Their requirements for this application are a high level of safety, low cost, low maintenance, and long cycle and calendar life. Specifically, for home-use photovoltaic systems, aqueous Na ion batteries seem to be the best choice in terms of sustainability, cost, and safety [1].

Aqueous Li ion rechargeable batteries have been under investigation over the last 25 years with the aim to find stable electrode materials in aqueous environments and avoid water electrolysis [2,3,4,5]. They satisfy the requirement of safety and cost because of the aqueous electrolyte. However, the sustainability concerns related to the use of Li, mainly for its limited abundance and geographic localization, remain unsolved, specifically when water in salt solutions are used [5].

Aqueous Na ion rechargeable batteries, in addition to the safety and cost requirement satisfied by the use of water as solvent, have Na as an ionic charge carrier that is 30 times cheaper and 1000 times more abundant than Li. These characteristics of Na are also important for attaining low-cost electrodes and salts [6].

Given the limited potential window of water-based electrolytes, the specific energy of aqueous Na ion batteries is low. However, the high conductivity of an aqueous electrolyte can provide high power [1].

Another aspect that affects the total cell cost is manufacturing. At present, the overall cell manufacturing represents ca. 50% of the cost of the cell [7]. This cost also includes the process to produce electrodes that consist of a deposition of a slurry of active materials on current collectors, evaporation of the organic solvent contained in the slurry, condensation of the solvent for its recovering and reuse, and final electrode drying. As already pointed out [8], the use of a toxic and high-boiling organic solvent for electrode preparation is the most common procedure. However, for moving toward more sustainable batteries, it is necessary to change from the beginning, starting from materials and processes. Several studies have been carried out on aqueous processes using water-processable binders [8,9,10,11]. Many of these binders come from natural sources or wastes, thus eliminating the need of using fluorinated binders that usually require toxic organic solvents, unless water-soluble fluorinated binders are used [12,13]. In any case, fluorinated polymers could be a problem at the end of the life of the battery. Some binders, such as sodium alginate, have been successfully used in Zn ion batteries. Zn^2+^ ions crosslink sodium alginate chains, forming a water-insoluble polymer structure that is stable in aqueous electrolytes with 10 M ZnCl_2_ and 4 M LiCl [14]. However, using water-soluble binders, such as carboxymethyl cellulose, sodium alginate, and other biopolymers could be not viable in other systems with aqueous electrolytes.

**Figure 1:**
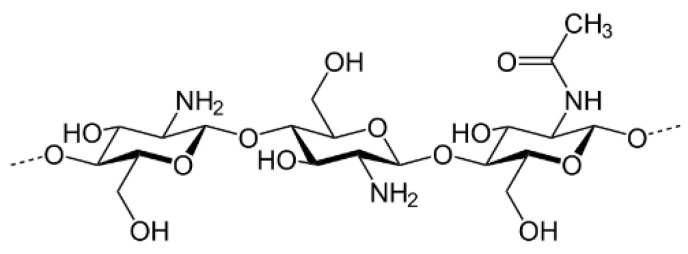
Chemical structure of chitosan.

Chitosan, which has already been studied as binder for nonaqueous Li ion batteries [15,16], is a linear polysaccharide composed of randomly distributed β-(1→4)-linked D-glucosamine (deacetylated unit) and N-acetyl-D-glucosamine (acetylated unit), obtained from crab and shrimp shells (Figure 1).

We demonstrated for the first time that crosslinked chitosan yields a stable and flexible structure for freestanding electrodes that can be used in aqueous Na ion cells.

The advantage of freestanding electrodes also relies on avoiding the use of current collectors, which is valuable in terms of use of raw materials, cost, battery weight, and volume. This is a preliminary study to prove a concept that can also be extended to other aqueous systems. Further studies are needed to optimize the crosslinking conditions, the electrode formulation, and the rheological properties of the slurry.

## 2. Materials and Methods

Carbon black C-45 (CB, Imerys Graphite & Carbon, Bironico, Switzerland), Picactif BP10 (PICA Co., Levallois-Perret, France), LiFePO_4_ (LFP, Aleees Co. Ltd., Taoyuan, Taiwan), NaH_2_PO_4_∙2H_2_O (Caesar & Loretz GmbH, Hilden, Germany), TiO_2_ (>99%, Sigma Aldrich, Merck KGaA, Darmstadt, Germany), (NH_4_)_2_HPO_4_ (>99%, Merck KGaA, Darmstadt, Germany), succinic acid (99.5%, Merck KGaA, Darmstadt, Germany), chitosan (>75% deacetylated from shrimp shells, Merck KGaA, Darmstadt, Germany), polytetrafluoroethylene (PTFE) suspension (solid content 60.2% *w*/*w*, DuPont de Nemours and Co. BV Nederland, Dordrecht, The Netherlands,), Na_2_SO_4_∙10H_2_O (99%, Merck KGaA, Darmstadt, Germany), Li_2_SO_4_∙H_2_O (ACS, Carlo Erba S.r.l., Milan, Italy), N-(3-Dimethylaminopropyl)-N′-ethylcarbodiimide hydrochloride (EDC, 98%, Merck KGaA, Darmstadt, Germany), 1 M LiPF_6_ in 1:1 (w:w) ethylene carbonate (EC): dimethyl carbonate (DMC) (LP30, 99.9%, Solvionic, Toulouse, France), and Graphite MAGE (D_50_ 22.8 µm, Hitachi Chemical Co. Ltd., Tokyo, Japan) were used without further purifications. Glass fibre membranes (GF/D, Whatman, GE Healthcare Ltd., Little Chalfont, UK) were used as separators.

### 2.1. Crosslinked Binder

**Figure 2:**
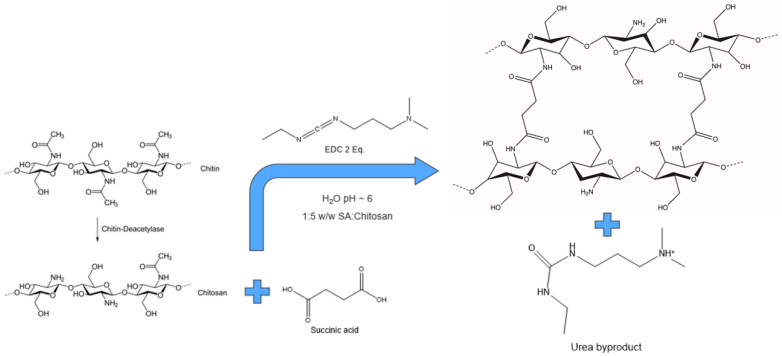
Process scheme of chitosan crosslinking.

Chitosan exhibits poor solubility in water and in most organic solvents because of strong intermolecular and intramolecular hydrogen bonding interactions [17]. Chitosan is soluble at pH values < 6.5 due to the protonation of the NH_2_ groups of the glucosamine units. For this reason, aqueous solutions of carboxylic acids dissolve chitosan more effectively [17,18]. Succinic acid and chitosan powders were added in water, in 1:5 *w*/*w*, and left under stirring for a couple of hours at room temperature until complete dissolution. The solution appeared like a gelatine. For crosslinking the chitosan chains, 2 equivalents of EDC for 1 equivalent of succinic acid were added to the solution. At least two equivalents of a crosslinking agent are required in order to form chemical bonds between all the amino groups, in chitosan chain, and the two carboxylic terminations of succinic acid, according to the stoichiometry (Figure 2).

In a short time, chitosan became a white solid, floating above the reaction solution. The solid was recovered and washed with water to remove reaction byproducts.

Although chitosan deposited via drop casting on mylar sheets formed flexible thin films, crosslinked chitosan samples became a hard solid mass. Chitosan deposited on mylar and crosslinked chitosan have been dried at RT overnight, and for 16 h at 80 °C before performing physicochemical analyses.

### 2.2. Electrode Materials Synthesis and Electrode Preparation 

#### 2.2.1. Na_2_Ti_2_(PO_4_)_3_ Synthesis

For sodium titanium phosphate (NTP) preparation, we followed the synthesis procedure described by Cao et al. [19]. The reaction leading to the formation of NTP is the following:

NaH_2_PO_4_∙2H_2_O + 2 TiO_2_ + 2 (NH_4_)_2_HPO_4_ → NaTi_2_(PO_4_)_3_ + 4 NH_3_ + 6 H_2_O

**Figure A1:**
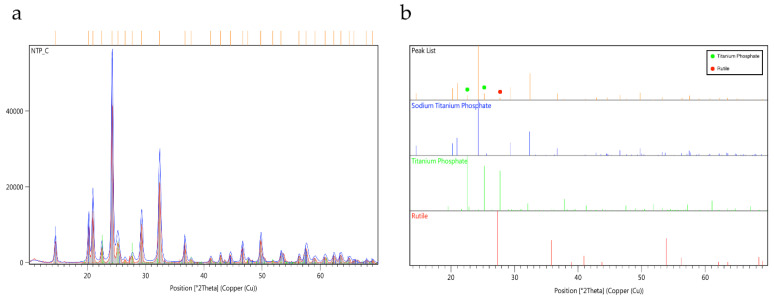
(**a**) XRD spectrum of NTP, (**b**) comparison of NTP peak list with reference compounds.

**Figure A2:**
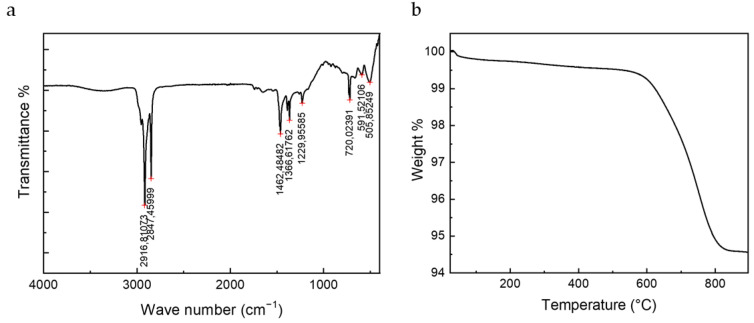
(**a**) FTIR spectrum of NTP and (**b**) TGA curve.

**Figure A3:**
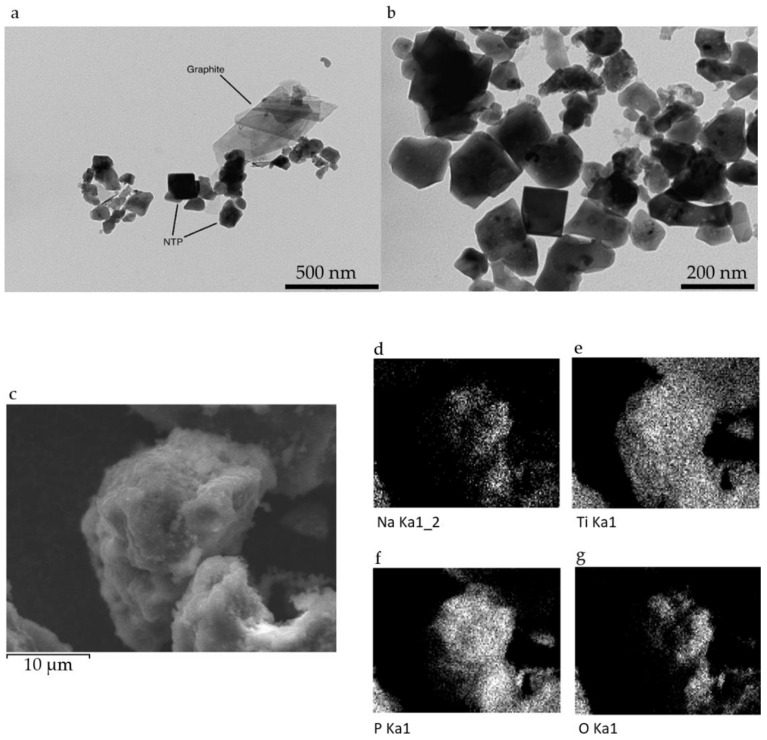
(**a**,**b**) TEM images of NTP powder, (**c**) SEM image of NTP and (**d**–**g**) elemental distribution of Na, Ti, P, and O, respectively.

The reactant powders were mixed in a stoichiometric ratio and ball milled for 10 min at 250 rpm using a tungsten carbide jar with 10 tungsten carbide balls (5 mm diam). Carbon black (1.5 wt.%) and graphite (2.5 wt.%) were then added and left under milling at 350 rpm for 60 min. The resulting intermediate product was ground to obtain a homogeneous powder and then thermally treated under argon atmosphere for 2 h, up to 700 °C with a thermal ramp of 20 °C/min [20]. The physicochemical and electron microscopy characterization of NTP is reported in Appendix A in Figure A1, Figure A2 and Figure A3.

#### 2.2.2. Electrode Preparation

**Figure 3:**
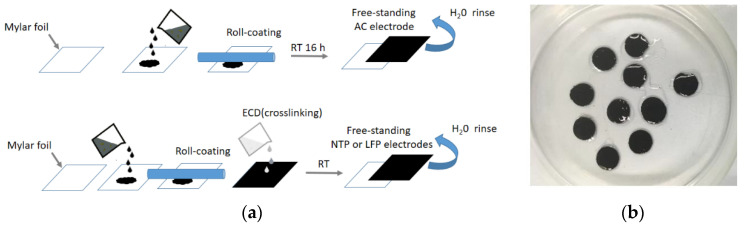
(**a**) Self standing electrode preparation scheme and (**b**) self-standing electrodes soaked with EDC solution.

Electrode preparation was carried out according to the scheme shown in Figure 3. Activated carbon (AC) electrodes were prepared for use as counter electrodes for the study of the NTP anodes. For activated carbon, the electrode slurry was prepared by mixing 85 wt.% active material (Picactif), 10 wt.% CB, and 5 wt.% of chitosan. Succinic acid and chitosan (1:5 *w*/*w*) have been dissolved under stirring in water. After the complete dissolution of the binder, active material and carbon conductive additive were added and left overnight under stirring. EDC was added and the solution was left under stirring for 12 h to allow crosslinking. The slurry was roll coated onto a mylar sheet. After drying at RT, the electrode sheet had been detached from the mylar, and self-standing electrodes have been cut and heat treated at 120 °C for 12 h. The obtained electrodes had a mass loading in the range of 4.6 and 5.0 mg/cm^2^.

For NTP and LFP, the electrode slurries were prepared by adding 80 wt.% active material (NTP or LFP), 10 wt.% CB and 10 wt.% chitosan. LFP electrodes were also prepared by using a lower amount of chitosan brought to electrodes breaking over cutting. The same steps described for AC electrodes preparation were followed, except the addition of EDC that was performed directly on each electrode. Indeed, the addition of EDC in the solutions produced agglomerates and it was not possible to roll coat the slurry on mylar. The electrodes deposited on mylar were soaked with 100 µL of 20 mM aqueous solution of EDC that contains approximately the stoichiometric amount of EDC with respect to the chitosan contained in the electrode disks. The reaction took place for 12 h, and then electrodes were washed with water, and dried at 120 °C overnight. Following the same manufacturing steps, we also prepared crosslinked LFP electrodes, with a different amount of EDC: 1, 0.5, and 0.1 equivalents respectively. The NTP electrodes had an active material mass loading in the range 5.8–6.5 mg/cm^2^, and the LFP electrodes 4.2–7.2 mg/cm^2^. The LFP electrodes prepared with 0.1, 0.5, and 1 equivalent of EDC had a mass loading of the active material in the range of 4.2–6.3 mg/cm^2^, respectively. LFP prepared with a PTFE binder had a LFP loading of 10.8–11.3 mg/cm^2^. With the same procedure, we also prepared AC electrodes (85:10:5 *w*/*w*) with the addition of the EDC solution on the electrode disks, by decreasing the crosslinking time to 4 h. We used two different EDC solutions: one without modifying the pH (ca. 6.0) and the other by decreasing the pH to 5 for a faster reaction of EDC as reported by Wrobel et al. [21]. The obtained electrodes had a mass loading in the range of 3.6–4.8 mg/cm^2^.

### 2.3. Physicochemical Characterization 

Functional group analysis for succinic acid, chitosan, and crosslinked chitosan was performed by Fourier transform infrared spectroscopy (FTIR). The samples were ground with KBr and the powder was compressed at 6 tons for 120 s. FTIR spectra were collected by using a Bruker Alpha with the resolution of 5 cm^–1^ in the range of 400–4000 cm^–1^. Thermogravimetric analysis (TGA) was performed by TA Instruments Q50 in O_2_ (sample gas) 60 mL/min for NTP analysis, and argon as a sample gas 60 mL/min for binder characterization. Argon (balance gas) 40 mL/min for both analyses was used. X-ray diffraction (XRD) spectra of powders and electrodes were carried out by Pan Analytical X’pert PRO with an X’Celerator detector (radiation Cu Ka, 40 mA, 40 kV), and scanning electron microscopy (SEM) images were collected by a Zeiss EVO 50 equipped with an Oxford INCA Energy 350 analyser. TEM images were collected by a Philips CM100 with 80 kV electron acceleration voltage. 

### 2.4. Electrochemical Characterization

Electrodes were characterized by cyclic voltammetry, galvanostatic charge/discharge cycles and impedance spectroscopy. Swagelok cells (three-electrode mode) and Biologic VSP potentiostat/galvanostat have been used for electrochemical tests. An Ag disk (−0.11 V vs. saturated calomel electrode, SCE) was used as a pseudoreference electrode, and the potentials were calculated vs. SCE.

## 3. Results and Discussion

### 3.1. Binder Characterization

In the present study, we have chosen a natural polymer to evaluate the possibility of using it as a water-soluble binder, to reduce the impact of electrode manufacturing processes, and to study the effect of chemical crosslinking to avoid binder dissolution in aqueous batteries [8,9]. It is known that acetic and formic acid are effective for the dissolution of chitosan in water. The “proton exchange” between –COOH groups of the mentioned acids and free –NH_2_ groups of chitosan could be the reason for the dissolution of chitosan in acidic solutions. Therefore, it has been expected that succinic acid is also able to donate protons and, hence, to dissolve chitosan [22]. Because of the proton exchange, chitosan was dissolved in the presence of succinic acid in water; therefore, the use of acetic or formic acid is avoided [23,24]. Besides human health risks, especially regarding concentrated formic acid [25], succinic acid’s role is twofold: to dissolve chitosan through proton exchange, and to provide, with the double carboxylic termination, the functional groups for chemically crosslinking the chitosan chains. Furthermore, succinic acid production is achieved not only from a petroleum refinery, but also from both anaerobic and aerobic processes from biomass fermentation [26].

**Figure A4:**
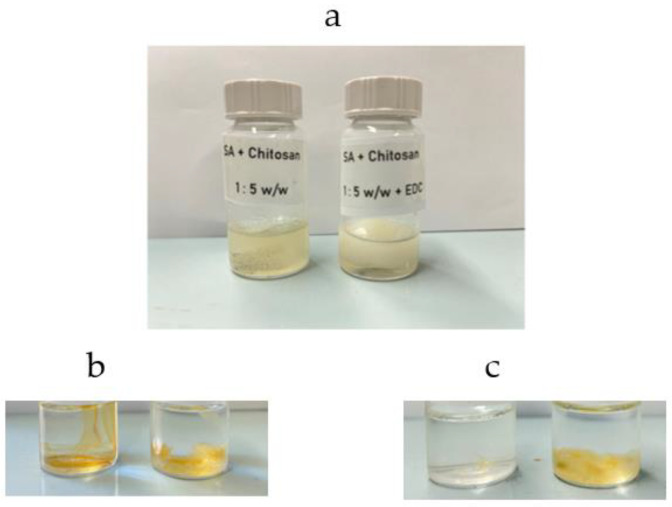
(**a**) Chitosan solution and formation of crosslinked chitosan; chitosan, and crosslinked chitosan membrane in water after (**b**) 7 days and (**c**) 30 days.

Having in mind the use of chitosan in aqueous batteries, we started with solubility tests, soaking chitosan and crosslinking chitosan in 10 mL of water. It can be seen that even after a short period of one week, chitosan dissolved, whereas the crosslinked chitosan was still visible in the water even after 4 weeks, as shown in Figure A4. 

**Figure 4:**
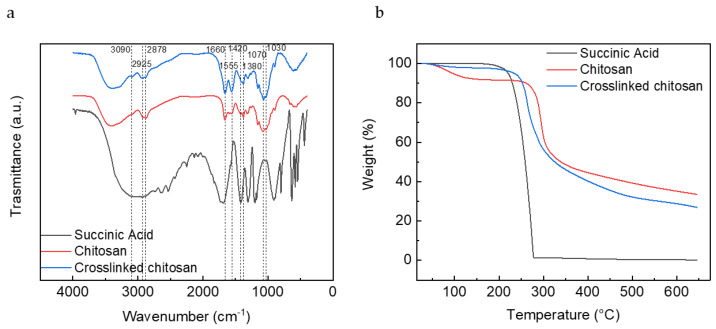
(**a**) IR spectra and (**b**) TGA curves of succinic acid, chitosan, and crosslinked chitosan.

The FT-IR spectra in Figure 4a shows the chemical modifications in chitosan structures upon interaction with succinic acid and EDC. There are no significant changes between chitosan and crosslinked chitosan spectra. The signals in the region between 2800 and 3000 cm^−1^, corresponding to ammonium ions, are slightly more defined in the spectra of the crosslinked chitosan. Absorption in 1661 cm^–1^ and 1555 cm^–1^ correspond to the presence of the asymmetric N–H bend and asymmetric COO^−^ stretching in amides, respectively. The peak observed at 1424 cm^–1^ and 1380 cm^–1^ was due to the symmetric N–H bend and symmetric stretching, and signals at 1070 cm^–1^ and 1030 cm^−1^, correspond to C–O stretching in acetamide. Other peaks observed in the crosslinked chitosan spectrum were similar to the native chitosan, demonstrating that there was no change in the main backbone of the chitosan structure.

TGA curves for the succinic acid, chitosan, and crosslinked chitosan samples are shown in Figure 4b. The incorporation of succinic acid in crosslinked chitosan tends to shift the thermal decomposition region of succinic acid to higher temperatures due to crosslinking. After complete decomposition of succinic acid, the curve of chitosan and the crosslinked sample maintain the same trend. It could be seen that even if the samples have been dried before the measurement, chitosan still retains some water, as shown by the mass decrease around 100 °C. 

**Figure A5:**
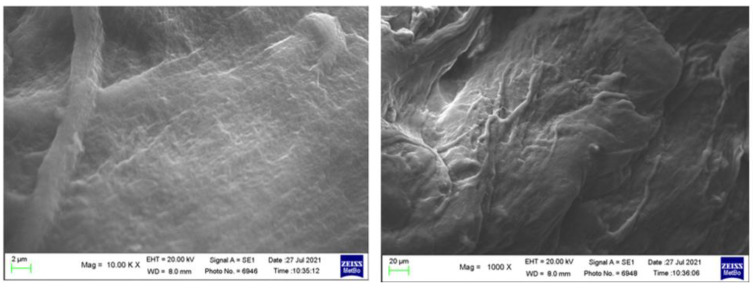
SEM images of crosslinked chitosan morphology at different magnification.

SEM images of crosslinked chitosan show its dense and compact mass, with visible filaments across the polymer structure as shown in Figure A5.

### 3.2. Electrode Preparation and Characterization

Several types of freestanding electrodes were prepared with NTP, LFP, and AC, the latter being used as a counter electrode in the electrochemical cells. CVs were performed in 1.5 M Na_2_SO_4_ and of 1 M Li_2_SO_4_ aqueous solution

**Figure 5:**
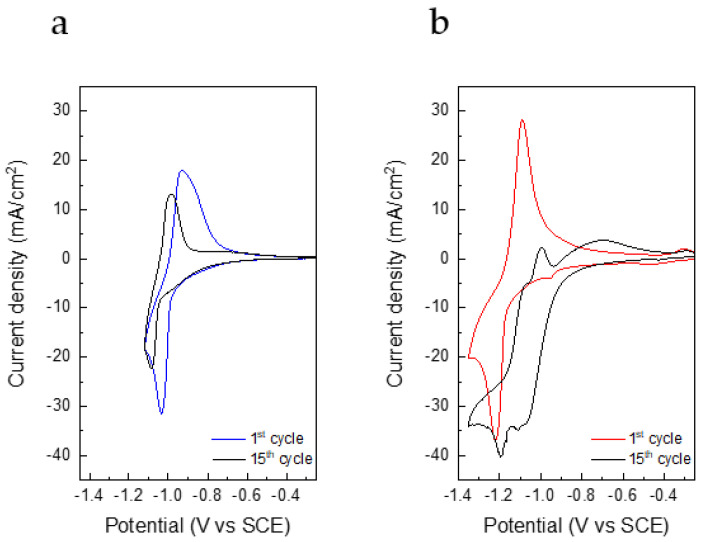
CV at 0.5 mV/s of NTP electrodes in (**a**) Na_2_SO_4_ and (**b**) Li_2_SO_4_.

**Figure A6:**
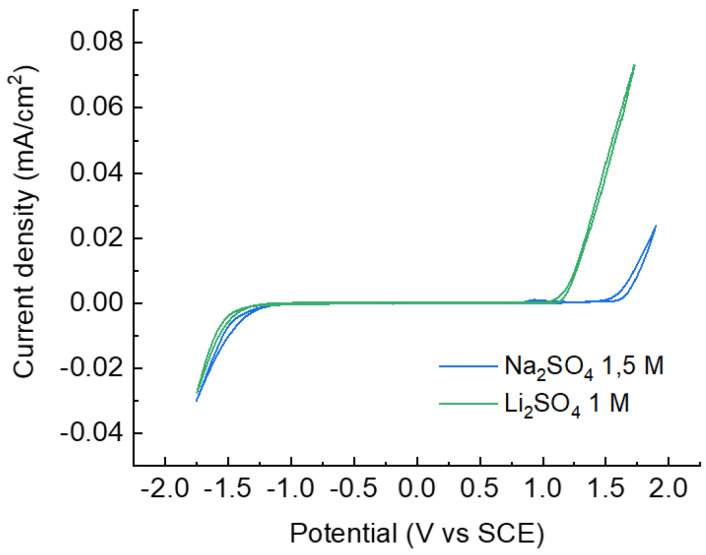
Cyclic voltammetry of different electrolyte solutions at 20 mV/s.

Figure 5 shows the CVs of NTP electrodes in the three electrolytes. NTP electrodes had the same electrochemical behaviour in all the electrolytes, showing the peaks of redox couple Ti^4+^/Ti^3+^ with the insertion of Na^+^ ions around −1.0 V vs. SCE and around –1.2 V vs. SCE with the insertion of Li^+^. However, even if the initial capacity values are good for aqueous systems, there is a progressive decrease in the current peaks. This could be due to the faradic process taking place at the edge of the electrochemical stability window where hydrogen evolution reaction starts. Figure A6 reports the background CVs of a stainless steel electrode in the two electrolytes. Furthermore, following the hydrogen reduction, the local concentration of OH^−^ ions increases, which is detrimental for the NTP that could release Ti(OH)_4_ and PO_4_^3−^ ions [27,28]. In Figure 5a, only one peak is present due to the insertion of Na^+^ ions, and after 15 cycles, a shift of both peaks is evident. In Figure 5b, the first cycle is related to the insertion of Li^+^, whereas in the subsequent cycles, the peaks related to the insertion of Na^+^ are also present [29]. This redox process corresponds to the progressive insertion and de-insertion of Li^+^ ions, as well of Na^+^ ions, which leads to the structural change of the active material from NaTi_2_(PO_4_)_3_ to LiTi_2_(PO_4_)_3_ [29].

**Figure 6:**
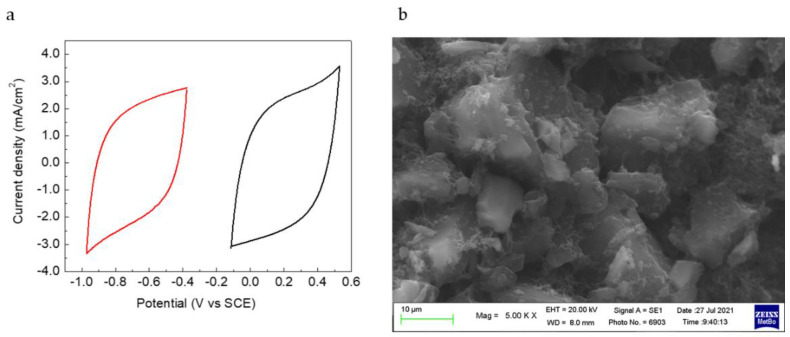
(**a**) CV curves of AC electrodes in Na_2_SO_4_ electrolyte and (**b**) SEM image of pristine AC electrodes surface.

To assess if the crosslinked binder is stable over electrode cycling, and given that the performance of the synthesized NTP is affected by the electrochemical stability window of the electrolyte [29], the activated carbons used as counter electrodes in the cell with NTP were also tested. The CVs of these electrodes in Na_2_SO_4_ display a typical capacitive behaviour. The material is stable in a wide range, nearly 1.5 V from −1.0 V to 0.5 V vs. SCE. Figure 6a displays the CVs of the negative and positive electrodes of an AC//AC cell vs. the Ag pseudoreference electrode, and Figure 6b, the SEM images of one pristine electrode.

**Figure 7:**
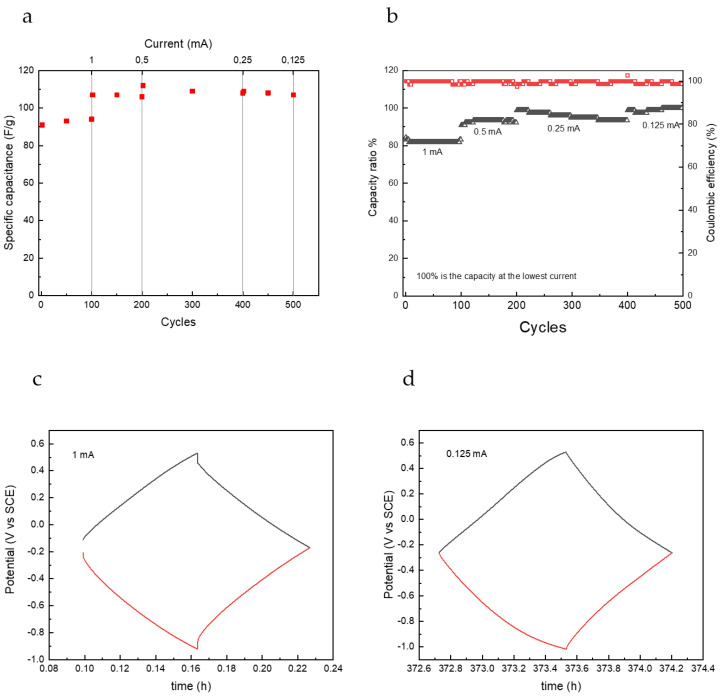
Performance of AC electrodes in AC//AC cell in Na_2_SO_4_ 1.5 M: (**a**) specific capacitance values, (**b**) coulombic efficiency and capacity ratio, voltage profiles of working electrode (black) and counter electrode (red) during galvanostatic charge and discharge cycles at (**c**) 1 mA and (**d**) 0.125 mA.

The stability of AC electrodes was also tested over 500 galvanostatic cycles with different charge/discharge current. Capacitance values shown in Figure 7a are quite interesting for this kind of capacitive electrodes operating in neutral aqueous electrolytes. These values are not greatly affected by the current rate in the range 0.2–0.05 A/g, remaining around 90–110 F/g. Fic et al. found specific capacitance of 130 F/g for activated carbon in 1 M Na_2_SO_4_ [30]. Coulombic efficiency values were very high during all cycles, and the capacity ratio, the ratio between the maximum capacity value achieved at the lowest current divided by each cycle capacity, is more than 80% at the highest current, as shown in Figure 7b. The electrode demonstrated a great stability in the aqueous environment and worked well either at high or low currents, as can be seen from the shape of charge and discharge curves in Figure 7c,d. At the lowest scan rate, a maximum specific capacity value of 26 mAh/g (0.14 mAh/cm^2^) was obtained, which is really good for a self-standing capacitive electrode in an aqueous, neutral environment. A value of ca. 34 mAh for g of active material in the single electrode has been reported for activated carbon fibres deposited on Ni foam in 6 M KOH [31].

**Figure 8:**
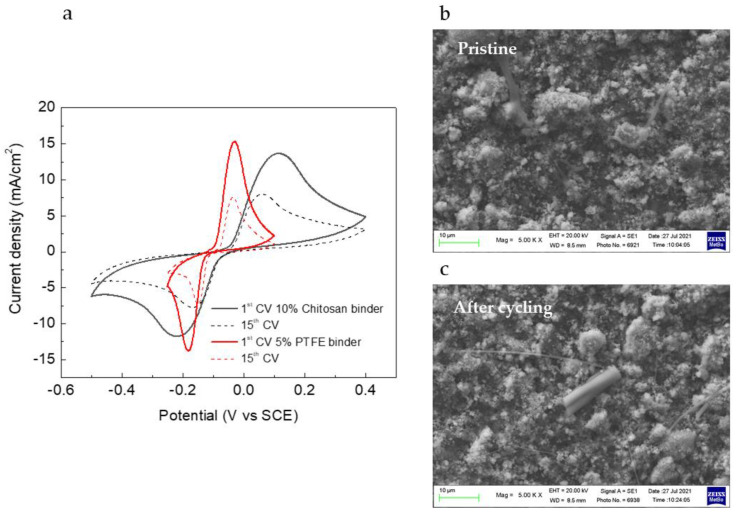
(**a**) CV curves of different LFP electrodes at 0.5 mV/s in 1 M Li_2_SO_4_ aqueous solution and (**b**,**c**) SEM images of pristine and cycled LFP electrodes.

LFP electrodes were prepared and tested in symmetric cells, after having fully charged the working electrode in another cell with the Li as a counter electrode. LFP electrodes were prepared with different binders, crosslinked chitosan, and PTFE aqueous suspension. The LFP//FP cells were tested in LiSO_4_ and in LP30 for comparison. Figure 8a shows the CVs of the LFP//FP in LiSO_4_ with the two binders. The current densities are almost the same for the electrodes with different binders, and the cycling behaviour is very similar. It is evident that the system with the crosslinked chitosan binder has more kinetic problems than the one with the PTFE binder, which could also be ascribed to the higher chitosan percentage content in the electrode formulation. However, in both cases, the same decrease in performance after 15 cycles was evident. SEM images of the LFP electrode with the chitosan binder are shown in Figure 8b, where the pristine electrodes and electrodes after CVs are compared. The decrease in performance cannot be ascribed to a loss of mechanical integrity of the electrodes, because no substantial differences could be seen between pristine and cycled electrodes. The superficial structure remains unchanged.

**Figure 9:**
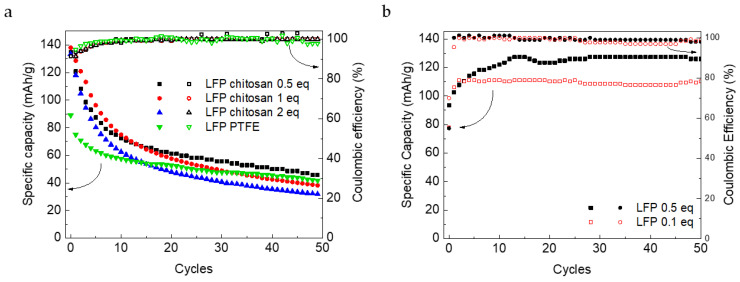
Specific capacity and coulombic efficiency value for LFP electrodes with different binders in LFP/FP cells: (**a**) chitosan crosslinked with 2, 1, and 0.5 equivalents of EDC and PTFE as a binder in 1 M Li_2_SO_4_ aqueous solution and (**b**) chitosan crosslinked with 0.5 and 0.1 equivalents of EDC in organic electrolyte (LP 30).

Figure 9 shows the galvanostatic charge and discharge cycles at a C-rate of C/2. The initial specific capacity values (133 mAh/g) are similar to those reported in literature for LFP in Li_2_SO_4_ solutions [32,33] and are specifically good for thick, self-standing electrodes operating, well higher than that of electrodes with the PTFE binder. However, the capacity retention, especially during the first 10 cycles, is poor, assessing into a final value around 40 mAh/g, similar to the value obtained with the PTFE electrode. Even if the solutions were de-aerated, the assembly of the cell was made in air and traces of oxygen could be present. It is known that dissolved oxygen gives some undesired reactions that lead to a capacity loss of LFP [34]. We tested the effect of crosslinker amount on this kind of electrode. Electrodes with a 1, 0.5, and 0.1 equivalent were tested by charge and discharge galvanostatic cycles. The electrodes in the aqueous electrolyte seem to be not greatly affected by the amount of crosslinker, either in terms of specific capacity or in terms of stability, as shown in Figure 9a. LFP electrodes with crosslinked chitosan have also been tested in LP30 for comparison, even if in organic electrolyte crosslinking is not necessary. For this reason, electrodes with the lowest amount of EDC (0.5 and 0.1 equivalents) were tested in LP30. Compared to the aqueous electrode, after the first cycles, the capacity values stabilized at 125 mAh/g, which is not far from those of commercial electrodes (150 mAh/g) [35]. An increase in specific capacity values over cycling, visible in Figure 9b, occurred due to a gradual wetting of the electrodes. 

**Figure A7:**
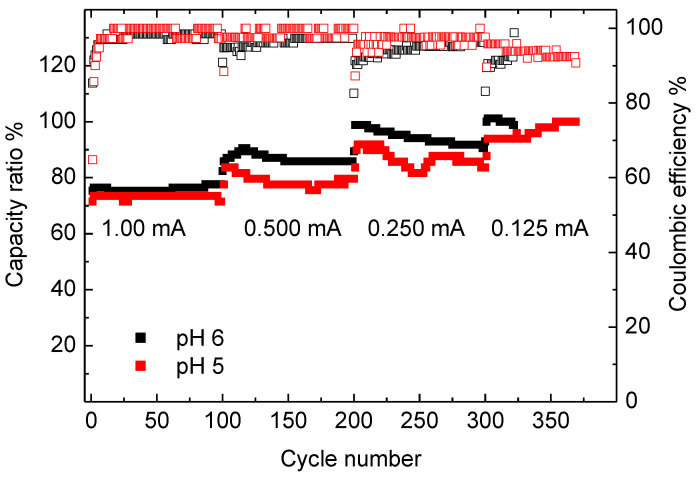
Performance of AC electrodes in AC//AC cell in Na_2_SO_4_ 1.5 M: coulombic efficiency and capacity ratio at 1.00 mA, 0.500 mA, 0.250 mA and 0.125 mA.

To evaluate the effectiveness of the crosslinking process, the same methodology applied to NTP and LFP electrodes was followed for AC electrodes. Electrodes were prepared in different conditions of crosslinking time and pH of the EDC solution (see Figure A7). From these experiments, it seems that decreasing the pH to 5 could improve the crosslinking process, making it possible to decrease the crosslinking time. The results are very similar to those reported in Figure 7b, even if the capacity and coulombic efficiency data are more scattered, evidencing that the addition of EDC in the slurry produces the best electrodes. Keeping in mind that the capacitive and faradaic charge/discharge processes affect the electrode structure in different ways, it cannot be excluded that the optimization of the crosslinking conditions, with the addition of surfactants to decrease particle aggregation [36] and improve the slurry viscosity, can eventually increase the stability of NTP and LFP electrodes. 

## 4. Conclusions

In this study, we investigated the use of chitosan as a sustainable binder, both in terms of material and electrode processing, for aqueous electrochemical storage systems. A successful crosslinked reaction involving chitosan and succinic acid, with the addition of a coupling agent, EDC, resulted in a water-insoluble binder. To our knowledge, it is the first time that such an approach is proposed for freestanding, thick electrodes for aqueous Na ion batteries. This concept can also be applied to other electrochemical energy storage systems. Indeed, we demonstrated that the crosslinked binder is stable over 500 galvanostatic cycles by using activated carbon electrodes, indicating the feasibility of this binder with capacitive and insertion systems in an aqueous environment. Indeed, we also demonstrated that the LFP electrodes with a crosslinked chitosan binder worked in the Li_2_SO_4_ electrolyte with initial capacity values near 130 mAh/g. The observed capacity fade could be due to O_2_ traces from the cell assembly in air, despite the use of de-aerated solutions. Indeed, the same electrode, even with a smaller amount of crosslinkers, is stable in an organic electrolyte and exhibits a capacity of 125 mAh/g. Another explanation of the capacity fade of NTP and LFP could be an ineffective binder crosslink in the whole electrode bulk. Further studies are needed to optimize the crosslinking conditions (binder and EDC amounts, pH, temperature, time, surfactant additives) in order to improve the electrochemical performance of the electrode with water-soluble chitosan for use in aqueous systems. 

## Data Availability

Not applicable.

## Appendix A

### Appendix A.1. Physicochemical Characterization of NaTi_2_(PO_4_)_3_ (NTP)

The crystalline structure of the synthesized NTP samples were characterized by powder X-ray diffractometry. All observed diffraction peaks, represented in Figure A1a, agree well with the standard pattern. From Figure A1b, a couple of peaks corresponding to titanium phosphate (TiP_2_O_7_) and rutile (TiO_2_), probably left from an unreacted species, can also be seen. The presence of TiO_2_ and TiP_2_O_7_ could raise some concern, especially for catalytic activity of some undesired processes for the first one and for lowering ionic conductivity for the second.

Phosphate ions have four vibrational modes corresponding to symmetric and asymmetric stretching and bending. In the IR spectrum in Figure A2a, stretching and bending peaks related to the O–P–O bonds, are 1230 and 591 cm^−1^, respectively, whereas the signal observed at 1462 cm^−1^ is related to C–H of graphite. The signal at 720 cm^−1^ could be ascribed to the Ti–O stretching of TiO_2_.

From the TGA analysis visible in Figure A2b, in the O_2_ atmosphere, it can be seen that until 570 °C NTP is stable, and from 570 to 830 °C, there is a thermal degradation of carbon to carbon dioxide. From the mass variation of the sample, we could observe a total amount of carbon, around 5%.

### Appendix A.2. Electron Microscopy Characterization of NaTi_2_(PO_4_)_3_ (NTP)

NaTi_2_(PO_4_)_3_ crystals dimension and surface morphology were characterized by transmission electron microscopy (TEM) and scanning electron microscopy (SEM). Figure A3a shows crystals of uniform size (150–200 nm), as can be seen in Figure A3b. Graphite foils are visible in Figure A3a. The graphite was added during synthesis in order to improve the electronic conductivity of the active material. Figure A3c displays SEM images of the morphology of NTP particles and in Figure A3d–g, the elemental distribution of Na, Ti, P, and O atoms in the powder. From the SEM images, we could see that the NTP tends to form agglomerate of dozens of micrometers, and the elemental distribution is homogeneous in the material.

### Appendix A.3. Solubility Tests

Figure A4a shows the two initial solutions of chitosan without and with EDC after 2 h under stirring. The formation of crosslinked chitosan is evident. The two solutions were brought to dryness in order to separate the solid chitosan and the solid crosslinked chitosan, which were further dried at 80 °C for 16 h. A colour change occurred from transparent white to brownish for both samples. Figure A4b,c show the solid chitosan and crosslinked chitosan immersed in 10 mL of water. After 7 days, it is possible to see the chitosan solubilisation, whereas even after 30 days, the crosslinked chitosan mass is stable, although it acquired a gel-like aspect on the surface.

### Appendix A.4. Chitosan SEM Images

The SEM images of crosslinked chitosan samples in Figure A5 show the dense and compact structure of the polymer, with visible thin filaments running across the surface.

### Appendix A.5. Electrochemical Stability Window of the Electrolytes

The electrochemical stability window of the electrolytes, shown in Figure A6, has been evaluated by cyclic voltammetry at 20 mV/s on a conventional electrochemical cell with a stainless steel working electrode (0.567 cm^2^). The counter electrode was Pt wire and the reference electrode was saturated calomel electrode (SCE). From background tests, the Li_2_SO_4_ solution exhibits the narrowest electrochemical stability window, whereas Na_2_SO_4_ has the largest one.

### Appendix A.6. Electrochemical Tests of AC Electrodes Prepared in Different Conditions

With the same procedure used for the preparation of NTP and LFP electrodes, we also prepared AC electrodes (85:10:5 *w*/*w*) with the addition of the EDC solution on the electrode disks, by decreasing the crosslinking time to 4 h. We used two different EDC solutions: one without modifying the pH (ca. 6.0) and the other by decreasing the pH to 5 for a faster reaction of EDC. The electrochemical stability of AC electrodes in 1.5 M Na_2_SO_4_ solution is reported in Figure A7. Coulombic efficiency values decreased at lower currents and the capacity ratio, the ratio between the maximum capacity value achieved at the lowest current divided by each cycle capacity, is between 70% and 80% at the highest current. These values are very similar to those reported in Figure 7b, although more scattered, indicating that the addition of EDC to the slurry is the best procedure, whenever is possible.
